# The association of cognitive behavioral intervention with caregiver burden and quality of life among family caregivers of cancer recurrence patients

**DOI:** 10.3389/fpsyt.2026.1904821

**Published:** 2026-10-15

**Authors:** Zhan Zhang, Han Zhou, Xiaoyu Guo, Bo Zheng

**Affiliations:** 1 Department of Thyroid and Breast Surgery, Wuhan No 1 Hospital, Wuhan, China; 2 Day care Treatment Center, Oncology Center, Renmin Hospital of Wuhan University, Wuhan, China; 3 Department of Medicine, Yangtze University, Jingzhou, China

**Keywords:** cancer recurrence, caregiver burden, cognitive behavioral intervention, family caregivers, quality of life

## Abstract

**Objective:**

This study aimed to evaluate the immediate and 3-month follow-up associations of cognitive behavioral intervention (CBI) on caregiver burden and quality of life (QOL) among family caregivers of patients with recurrent cancer.

**Methods:**

A quasi-experimental study was conducted among 87 family caregivers of patients with recurrent cancer who completed the study at a tertiary hospital in China. Participants were recruited from two inpatient wards within the same oncology department, with one ward assigned to the intervention arm (n=43) and the other to the control arm (n=44). The intervention group received an 8-week structured CBI alongside routine care, while the control group received routine care alone. Outcomes were measured using the Zarit Burden Interview (ZBI) and the World Health Organization Quality of Life-BREF-Chinese Version (WHOQOL-BREF-CV), which were administered at baseline (T_0_), immediately post-intervention (T_1_), and 3-month post-intervention follow-up (T_2_).

**Results:**

Compared with the control group, the intervention group showed significant reductions in total caregiver burden at both T_1_ (MD = -8.27, 95% CI: -10.39 to -6.16; *d* = -1.66) and T_2_ (MD = -4.66, 95% CI: -6.92 to -2.40; *d* = -0.88). Significant improvements were also observed in overall QOL at T_1_ (MD = 3.39, 95% CI: 1.46 to 5.32; *d* = 0.75) and T_2_ (MD = 3.91, 95% CI: 1.83 to 5.99; *d* = 0.80), as well as in the physical domain at T_1_ and T_2_, and in the psychological domain at T_1_. However, the psychological-domain difference at T_2_ was less robust in *post hoc* multiplicity-adjusted sensitivity analyses. However, between-group differences in the social relationships and environmental domains were not statistically significant at T_2_ (*p* > 0.05). The control group showed no significant longitudinal changes in caregiver burden; however, significant within-group fluctuations were observed in several QOL domains over time.

**Conclusions:**

The findings suggest that CBI may be associated with reduced caregiver burden and improved QOL among family caregivers of patients with recurrent cancer over the immediate and 3-month follow-up period. Further large-scale, multi-center studies with extended follow-up are recommended to confirm the sustainability of these effects and the generalizability of the intervention.

## Introduction

Cancer remains a major global public health and economic burden. In 2022, there were an estimated 20 million new cancer cases and nearly 10 million cancer-related deaths worldwide (1). Notably, China accounted for over one-fifth of the global cancer incidence and mortality, representing the highest disease burden worldwide (2). Although advances in cancer treatments have substantially improved survival rates—particularly for breast and prostate cancers—prolonged survival is frequently accompanied by a high risk of cancer recurrence. Recurrence triggers a cascade of physical and psychological distress symptoms in patients, such as pain, nausea, anxiety, and fear (3). Consequently, this challenging trajectory places an immense psychological and caregiving burden on family caregivers (4). Specifically, while providing continuous physical and emotional support, family caregivers face profound psychological distress related to the uncertainties of disease progression and fear of death (5). Additionally, the substantial time and energy required for caregiving often restrict caregivers’ social activities and employment opportunities. This leads to diminished social networks and financial toxicity, which can further exacerbate their anxiety (6, 7). Ultimately, these compounding stressors severely compromise caregivers’ quality of life (QOL) (8–10). However, existing evidence suggests that alleviating caregivers’ burdens—both financial and psychological—can significantly improve their overall well-being (11).

Grounded by Beck’s cognitive model, cognitive behavioral intervention (CBI) is a structured psychological approach that combines cognitive restructuring with behavioral modifications (12, 13). It aims to help individuals identify and reappraise maladaptive thoughts, regulate emotions responses, and develop more adaptive coping behaviors (14). According to the cognitive model, emotional and behavioral responses are influenced not only by stressful events themselves but also by individuals’ interpretations of those events. In the context of cancer recurrence, caregivers may develop negative automatic thoughts, such as believing that they must manage all caregiving tasks perfectly, that recurrence reflects a failure of previous care, or that they are personally responsible for preventing further deterioration. These cognitions may intensify guilt, helplessness, anxiety, and perceived responsibility, thereby increasing personal and role strain (15). Emotional distress may also contribute to sleep disruption, withdrawal from social activities, reduced self-care, and ineffective problem-solving, which may further impair physical and psychological QOL (8–10, 16). The intervention was therefore designed to interrupt this reciprocal cycle of maladaptive cognitions, emotional distress, and unhelpful behaviors. Cognitive restructuring was intended to help caregivers identify and reappraise excessive responsibility and catastrophic interpretations; emotion-regulation and relaxation techniques were intended to reduce psychological and physiological arousal; problem-solving, time-management, and behavioral-activation strategies were intended to improve coping, restore self-care activities, and reduce role strain; and communication training was intended to facilitate support-seeking and improve interactions with patients, family members, and healthcare professionals (13, 16–20). Through these pathways, CBI may be associated with reduced caregiver burden and improved QOL among caregivers confronting cancer recurrence (16, 17). Nevertheless, evidence regarding CBI for family caregivers of patients with cancer remains mixed. A meta-analysis of CBI-based interventions found reductions in caregivers’ anxiety and depression, but no statistically significant improvement in quality of life, and highlighted the small number and methodological heterogeneity of available studies (17). Most previous interventions also included caregivers from different stages of the cancer trajectory or focused on particular cancer types, making it difficult to determine whether their findings apply to caregivers confronting confirmed recurrence (16). Caregiving after confirmed recurrence may differ from caregiving at both initial diagnosis and advanced disease. At initial diagnosis, caregivers primarily face the unfamiliarity of cancer, treatment decisions, and the initial adjustment to a caregiving role. By contrast, recurrence occurs after a period of remission or relative disease stability and may disrupt restored expectations of recovery, reactivate memories of previous treatment-related distress, and renew caregiving responsibilities. Advanced cancer caregiving is often dominated by progressive symptom burden, functional decline, palliative care needs, and end-of-life decision-making (21), whereas recurrent cancer represents a broader clinical transition that is not necessarily synonymous with terminal or advanced disease. Consequently, caregivers confronting recurrence may experience a distinctive combination of renewed uncertainty, diminished confidence in treatment, and fear of further progression. Recurrence-related concerns are common among family caregivers and are associated with greater anxiety, depression, and poorer quality of life (15). However, structured CBI has rarely been evaluated specifically among caregivers after a patient’s confirmed cancer recurrence. The Chinese cultural context may further shape caregivers’ responses to psychological interventions. Filial piety and family responsibility can provide caregivers with meaning, commitment, and motivation to continue providing care. However, strong perceived obligations may also contribute to self-imposed responsibility, concern about inadequate caregiving performance, reluctance to disclose distress, and reduced help-seeking (22, 23). A empirical study among Chinese caregivers has shown that filial values may function as either a psychological resource or a source of burden, depending on how caregiving obligations are interpreted and balanced with caregivers’ own needs (24). In Chinese oncology settings, diagnosis disclosure, family communication, and social support have also been associated with caregiver burden, suggesting that culturally shaped family roles and communication practices may influence both caregiver outcomes and engagement with interventions (25). Therefore, a CBI delivered in this context may need to acknowledge family obligation while reframing excessive responsibility, legitimizing self-care, and encouraging support-seeking. To this end, we designed a quasi-experimental study to evaluate the immediate and 3-month follow-up associations of CBI with caregiver burden and quality of life among family caregivers of patients with recurrent cancer in a Chinese hospital, thereby providing preliminary evidence for its potential benefits in the Chinese context.

## Methods

### Study design and participants

This quasi-experimental study was conducted from June 30, 2024, to January 25, 2025. A convenience sampling method was used to recruit participants from the Department of Oncology, Renmin Hospital of Wuhan University in Wuhan, Hubei Province, China. The inclusion criteria were as follows: (1) aged ≥18 years; (2) care for patients with clinically confirmed recurrent cancer (recurrent cancer is defined as the re-emergence of clinically detectable malignant disease following a documented period of complete remission after initial therapy); (3) ZBI score≥21; (4) taking primary responsibility for patient care; and (5) having clear consciousness and good reading and comprehension skills. The exclusion criteria were: (1) having severe physical or psychiatric comorbidities that could significantly affect their quality of life or ability to participate; and (2) being paid caregivers (e.g., hired domestic workers).

### Ethical statement

The study was conducted in accordance with the Declaration of Helsinki and was approved by the Ethics Committee of Renmin Hospital of Wuhan University in Wuhan, Hubei Province, China (IRB No.WDRY2024-K021). Written informed consent was obtained from all participants, and their confidentiality and anonymity were strictly maintained throughout the study.

### Sample size calculation

The sample size was calculated using PASS 20 software, with the post-intervention total caregiver burden score as the primary endpoint. Based on our pilot data (unpublished, n=20, 10 per group, conducted from March to May 2024 at the same hospital), the post-intervention total care burden scores were 44.31 ± 6.91 (control group) and 38.92 ± 6.91 (intervention group). Using a two-independent-sample t-test, with the statistical power set to 0.90 and alpha to 0.05, the required total sample size was calculated to be 72 participants, 36 per group. Taking into account an estimated 20% attrition rate, the target total sample size was finally confirmed to be 90 (45 per groups). During the recruitment process, 2 participants in the intervention group and 1 in the control group withdrew from the study; thus, 87 participants were eventually enrolled and analyzed, with 43 in the intervention group and 44 in the control group. Although the primary longitudinal analysis used a 2 × 3 repeated-measures ANOVA, the sample size was calculated based on the post-intervention between-group comparison (T_1_) using a two-independent-sample t-test, as this contrast represented the most relevant between-group comparison for estimating the observed group difference and required the least number of assumptions regarding the correlation structure of repeated measurements. This approach is conservative, as repeated-measures ANOVA generally provides greater statistical power for detecting group × time interactions than a single cross-sectional comparison. Nevertheless, we acknowledge that the sample size was not formally powered for the group × time interaction effect specifically.

### Procedure

Participants meeting the inclusion criteria were informed about the study’s purpose and procedures. After providing written informed consent, caregivers completed the initial assessment using the ZBI and QOL scales with assistance from the research team. Caregivers scoring less than 21 on the ZBI were excluded, while those scoring ≥ 21 were formally enrolled in the study.

Participants were recruited from two inpatient wards within the same oncology department. Before recruitment, one ward was designated as the intervention ward and the other as the control ward; therefore, allocation was ward-based with one ward per study arm. Ward-based allocation was used instead of individual randomization to reduce the risk of contamination of intervention content between caregivers. The two wards were located on separate floors within the same oncology department. Intervention sessions were conducted in a designated counseling room, and participants were reminded not to discuss intervention content with individuals from other wards. Despite these precautions, complete prevention of contamination could not be guaranteed. Both wards were managed by the same oncology department and followed the same institutional procedures for cancer treatment, nursing care, caregiver education, and routine clinical management. The control group received routine care provided by the oncology department. Routine care was delivered by registered oncology nurses in the control ward according to the department’s standard nursing-care procedures. Because routine care was embedded in standard clinical nursing rounds rather than delivered as a scheduled intervention session, its duration and frequency were not fixed and varied according to caregivers’ questions, patients’ clinical condition, and routine nursing needs. The content primarily included information on recurrent cancer, treatment procedures, medication guidance, symptom monitoring, nutritional support, management of treatment-related adverse effects, and basic caregiving precautions. To ensure consistency, nurses followed the same ward-level health-education protocol and routine nursing-care procedures. Caregivers were encouraged to ask questions during routine nursing rounds, and individualized explanations were provided when necessary. No structured psychological intervention, Cognitive Behavioral training, relaxation training, communication skills training, or scheduled group sessions were provided to caregivers in the control group.

The intervention team consisted of 2 psychiatrists, 2 clinical physicians, 3 oncology nurses, and 2 graduate students in nursing. To ensure consistency and standardization, all team members underwent unified training prior to the study. The CBI program consisted of eight weekly, face-to-face group sessions (5–8 participants per group), each lasting approximately 40 minutes. All sessions were conducted in a designated counseling room within the oncology department to ensure privacy and minimize external interruptions. Sessions were led by trained members of the intervention team, including psychiatrists and oncology nurses, depending on the session content.

As shown in **Figure 1**, we developed an original conceptual diagram for the present study. Conceptually informed by Beck’s cognitive model and standard cognitive behavioral therapy principles, this diagram illustrates how the CBI protocol was designed to interrupt the cycle of negative automatic thoughts, emotional distress, and maladaptive coping behaviors among caregivers in the context of cancer recurrence. Key CBI techniques integrated into the protocol included: (1) Identification of Automatic Thoughts: Helping caregivers recognize rapid, spontaneous cognitions occurring without deliberate rational analysis, which significantly influence emotional states. (2) Cognitive Restructuring: Facilitating the recognition and modification of maladaptive thought patterns by promoting the development of realistic and adaptive cognitions, alongside identifying alternative responses to recurrent stressors. (3) Behavioral Training: Providing systematic instruction in relaxation strategies (e.g., guided imagery, diaphragmatic breathing) to enhance physiological self-regulation. The CBI program was implemented through weekly face-to-face sessions, each lasting approximately 40 minutes. All sessions were conducted in a designated counseling room within the oncology department to ensure privacy and minimize external interruptions. The intervention was delivered in small, face-to-face groups, with each group comprising 5–8 participants. Sessions were led by trained members of the intervention team, including psychiatrists and oncology nurses, depending on the session content. Intervention dose was recorded as the number of sessions attended, and homework completion was documented by the session facilitator using structured checklists.

**Figure 1:**
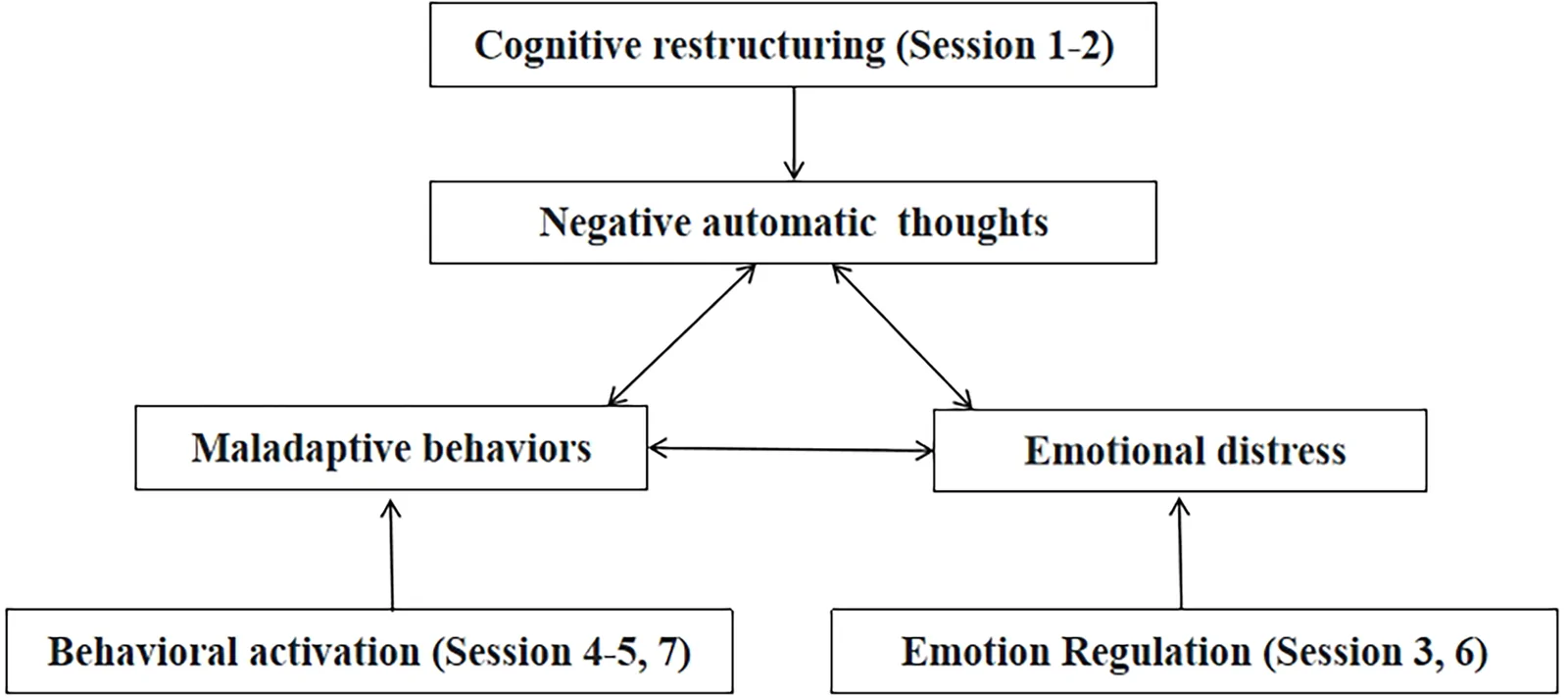
Conceptual diagram of the CBI framework.

Each session followed a standardized structure comprising four components: psychoeducation, cognitive or behavioral skills training, guided practice, and feedback. Psychoeducation introduced core CBI concepts tailored to caregiving stress, such as emotional labeling and negative automatic thoughts. Skills training covered techniques including automatic thought identification, cognitive restructuring, problem-solving strategies, behavioral activation, relaxation training, and communication enhancement. Guided in-session exercises, such as thought logs, relaxation drills, and scenario-based role plays, reinforced understanding, while real-life application was encouraged through structured homework assignments after each session.

Participants were provided with written materials and asked to complete brief daily logs of stressful caregiving experiences, thought responses, and coping strategies. These materials were reviewed and discussed during the subsequent session. Adherence to the intervention was monitored through attendance checklists and weekly team briefings. Intervention fidelity was maintained through ongoing supervision by the principal investigator and lead psychiatrist. Any deviations or protocol difficulties were resolved through weekly coordination meetings. Throughout the intervention, participants’ psychological well-being was monitored by session facilitators through observation and informal check-ins at the beginning and end of each session. Participants were encouraged to report any emotional discomfort during or between sessions. Facilitators provided immediate supportive counseling when needed, with referral to study psychiatrists available if clinically indicated. The specific intervention contents are detailed in **Table 1**.

**Table 1:** Specific intervention details.

Time	Theme	Core content	Implementers
Session 1	*Alliance Building*	1. Establish therapeutic alliance 2. CBI psychoeducation (Psychoeducation: Cognitive triad (Thought-Emotion-Behavior interaction))	Oncology nurse
Session 2	*Automatic Thought Identification*	1. Recognize automatic thought (AT) characteristics 2. identify negative automatic thoughts (e.g., "I must care for the patient perfectly").	psychiatrist
Session 3	Emotion Regulation: Acceptance & Awareness	1. Practice "emotion labeling" (e.g., anger, guilt, helplessness) 2. Establish situation-thought-emotion links("When ____happened, I thought ____")	psychiatrist
Session 4	Stress Management: Problem-Solving Training	1. Deconstruct caregiving stressors (e.g., medical communication, daily care, financial burdens). 2. Role-play scenarios (e.g., "How to communicate patient symptoms effectively with doctors").	psychiatrist
Session 5	Behavioral Activation: Self-Care Planning	1. Develop a "Self-Care Menu" (e.g., 15-minute walks, phone calls with friends). 2. Address "caregiver guilt" by distinguishing between "must-do" and "manageable" tasks through values clarification.	psychiatrist
Session 6	Relaxation Training	1. Diaphragmatic breathing (4-7–8 rhythm) 2. Progressive Muscle Relaxation (PMR)	Oncology nurse
Session 7	Social Support: Communication	1. Analyze communication patterns with patients and family members. 2. Practice "I-message" expression (e.g., "When you refuse medication, I feel anxious because I worry about your health").	Oncology nurse
Session 8	Summarize & maintenance	Establish long-term management	Oncology nurse

### Instruments

#### Demographic characteristics

The demographic questionnaire was developed by the research team and designed to comprehensively capture both caregiver - specific and patient - related demographic information. For caregivers, it included variables such as age, gender, relationship to the patient, marital status, religious affiliation, educational attainment, average daily caregiving hours, history of chronic diseases, and per capita monthly household income. Patient-related data collected via this questionnaire cover age, gender, self-care ability, primary disease diagnosis, type of medical insurance coverage, and level of disease awareness.

#### Zarit caregiver burden interview-Chinese version

Zarit first proposed the definition of caregiver burden and developed an assessment tool to measure caregivers’ perceived burden, known as the Zarit Caregiver Burden Interview (ZBI) (26). The Chinese version consists of 22 items, divided into two dimensions: personal strain and role strain. Responses are scored on a 5-point Likert scale (0~4) based on the degree of agreement, comprehensively assessing the caregiver’s physical health, psychological well-being, social life, financial status, and relationship with the patient. The total score categorizes burden into none/mild (≤20), moderate (21~39), and severe (≥40).The Cronbach’s α coefficient of ZBI-CV demonstrates a reliability of approximately 0.875. The ZBI has demonstrated strong validity among caregivers of patients with cancer (27).

#### World health organization quality of life-BREF-Chinese version

The WHOQOL-BREF is an abbreviated version derived from the original WHOQOL-100 (28). It comprises two items assessing overall quality of life and general health, along with one item from each of the 24 facets included in the WHOQOL-100. The WHOQOL-BREF evaluates four primary domains: physical health, psychological well-being, social relationships, and environment. The scale consists of 26 items, each rated on a 5-point Likert scale. In this study, the raw domain scores are reported, with each domain ranging from 4 to 20; higher scores indicate better perceived quality of life. The raw scores were used for all analyses and are presented in the results tables. A higher total scores indicates better perceived quality of life in the respondent. The Cronbach’s α coefficient for the international versions ranges between 0.75 and 0.89 (29), while the Chinese version demonstrates high reliability, with an α of approximately 0.90 (30).

### Data collection

Participants were screened for eligibility according to predefined inclusion and exclusion criteria and provided written informed consent prior to study initiation. Baseline (T_0_), post-intervention (T_1_), and 3-month follow-up (T_2_) assessments were administered by trained oncology nurses using the same standardized assessment procedures. Baseline and post-intervention assessments were conducted through face-to-face interviews. The follow-up assessment was performed in person whenever feasible. Otherwise, questionnaires were administered via WeChat video call following the same standardized protocol. Because of the ward-based allocation and the nature of the intervention, outcome assessors were not blinded to group allocation.

### Data analysis

The data in this study were processed and analyzed by statistical software SPSS 28.0 and R 4.3. Categorical variables were summarized as frequencies and percentages, and between-group differences were assessed using chi-square tests or Fisher’s exact tests, as appropriate. Continuous variables were summarized as means and standard deviations. Baseline differences between the intervention and control groups in caregiver burden and QOL outcomes were examined using independent-samples t-tests. Because caregiver burden and QOL were continuous outcomes measured repeatedly in the same participants at three prespecified time points, a 2 (group) × 3 (time) repeated-measures analysis of variance (ANOVA) was conducted, with group as the between-subjects factor and time as the within-subjects factor. The main effects of group and time and the group-by-time interaction were examined. Before the main analyses, the assumptions underlying repeated-measures ANOVA were assessed. Sphericity was evaluated using Mauchly’s test; when the assumption was violated, Greenhouse–Geisser-corrected degrees of freedom and p-values were reported for the time and group-by-time interaction effects. Normality of residuals within each group-by-time cell was assessed using the Shapiro–Wilk test, and homogeneity of variance between groups at each time point was evaluated using Levene’s test. When a statistically significant group-by-time interaction was detected, simple-effects analyses were performed. Between-group comparisons at each time point were conducted using independent-samples t-tests or Welch’s t-tests when the homogeneity-of-variance assumption was violated. Within-group pairwise comparisons across time points were adjusted using the Bonferroni correction. Effect sizes for the repeated-measures ANOVA were reported as partial eta squared (η²*p*), with values of 0.01, 0.06, and 0.14 interpreted as small, medium, and large effects, respectively. The primary analysis was conducted according to the per-protocol principle using complete cases. Participants who withdrew from the study or did not complete all scheduled outcome assessments were excluded from the primary analysis, and missing outcome data were not imputed. To assess the robustness of the repeated-measures ANOVA findings, a linear mixed-effects model was fitted as a sensitivity analysis for each outcome using the same per-protocol complete-case sample as the primary analysis. The model included group, time, and the group × time interaction as fixed effects and participant as a random intercept, and was estimated using restricted maximum likelihood. Participants with missing outcome observations were not included in the LMM sensitivity analysis, and missing data were not imputed. However, because the intervention was delivered in small groups (5–8 participants per group with shared facilitators), clustering at the intervention group level may have been present. The sensitivity model did not include session-group or facilitator random effects, as the study was not designed or powered to estimate these effects reliably (the intervention arm was the only arm delivered in a group format, and the number of groups was limited). Consequently, standard errors may be underestimated, and the findings should be interpreted with this limitation in mind. Statistical significance was set at a two-sided *p* < 0.05. To address multiplicity, *post hoc* multiplicity-adjusted sensitivity analyses were conducted using the Benjamini-Hochberg false discovery rate (FDR), Holm, and Bonferroni methods. These analyses were not prespecified in the original statistical analysis plan and were used to examine the robustness of the findings rather than as the basis for the primary conclusions. For this *post hoc* sensitivity assessment, comparisons were organized into three families: Family A included the group × time interaction tests for the eight outcome measures; Family B included the between-group comparisons at T_1_ and T_2_ across the eight outcome measures; and Family C included the within-group pairwise comparisons across time points. These family definitions were not prespecified. Multiplicity-adjusted results are presented in **Supplementary Tables 1**, **2**.

Of the 43 participants in the intervention group included in the final analysis, the overall attendance rate was 95%, corresponding to a mean of 7.6 out of 8 sessions attended. In this study, full intervention attendance was defined as attendance at all eight sessions, whereas adequate intervention dose was defined as attendance at ≥ 6 of the 8 sessions. A total of 41 participants (95.3%) attended at least 6 sessions, which was considered an adequate intervention dose. Therefore, not all participants attended the full eight-session program. The most frequently cited reasons for missed sessions were scheduling conflicts (n=2), deterioration of the patient’s condition (n=1), and caregiver illness (n=1). The homework completion rate was 94%, corresponding to a mean of 7.5 out of 8 weekly assignments completed. A total of 40 participants (93.0%) completed at least 5 of the 8 homework assignments. These data indicate satisfactory participant adherence and engagement with the intervention protocol.

## Results

### Demographic characteristics of participants

The participant flow through the study is shown in **Figure 2**. A total of 90 family caregivers met the inclusion criteria, consented to participate, and completed the baseline assessment. During the intervention period, one participant in the intervention group withdrew due to scheduling conflicts, and another was lost to follow-up due to patient a hospital transfer. In the control group, one participant was lost contact for unknown reasons. No patient deaths occurred during the study period; therefore, patient death did not contribute to participant attrition. Consequently, 43 participants in the intervention group and 44 in the control group completed all scheduled study assessments through T_2_ and were included in the final analysis. There were no statistically significant differences in the baseline demographic and clinical characteristics of either the caregivers or the patients between the two groups (*p* > 0.05), indicating baseline homogeneity. No adverse psychological events were reported or observed among the 43 intervention-group participants included in the final analysis throughout the 8-week intervention period (**Table 2**).

**Figure 2:**
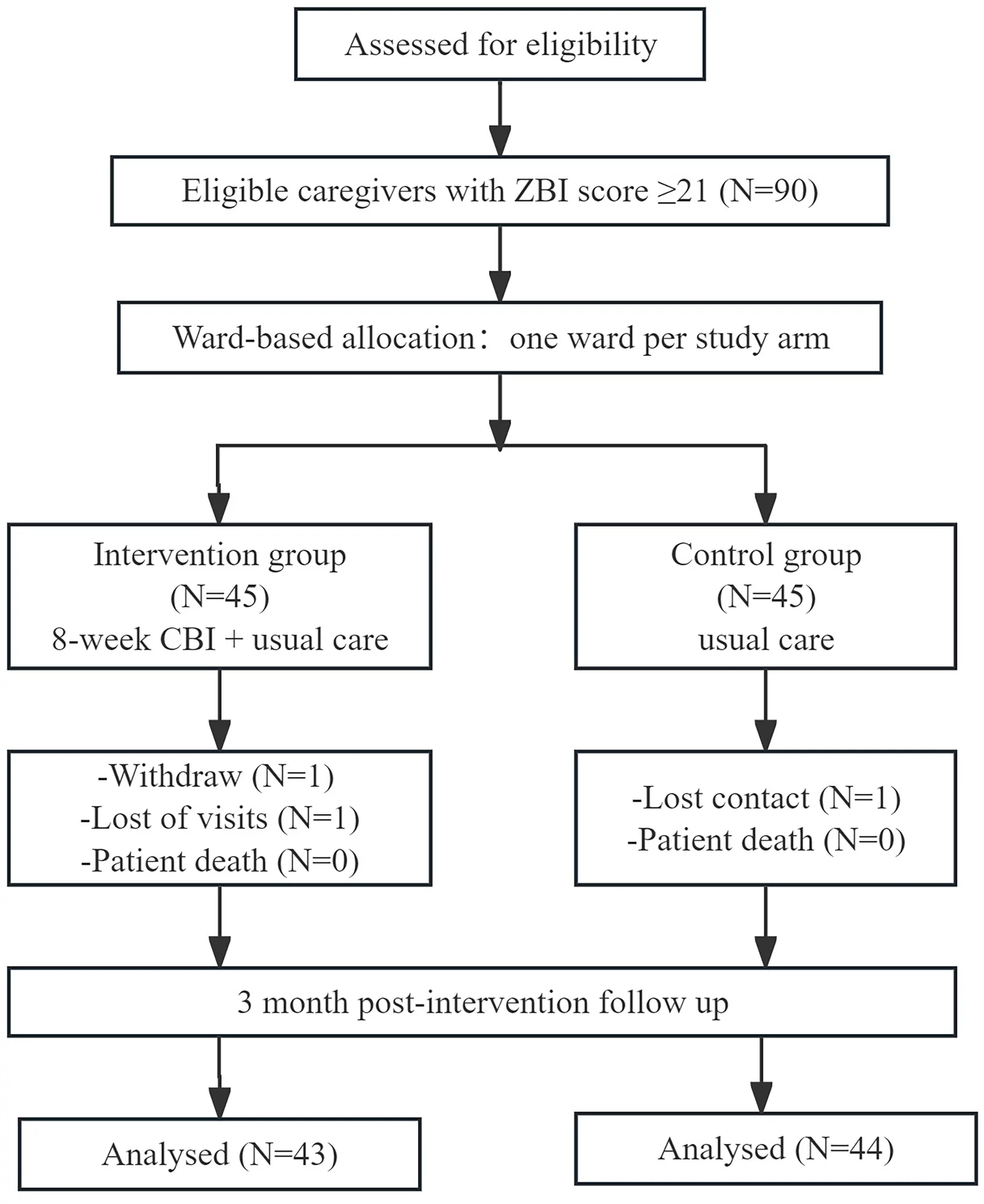
Participants flow diagram.

**Table 2:** Participant demographic characteristics at baseline.

Characteristics	Control group (n=44)	Intervention group (n=43)	*χ²*	*p*
Caregiver Age [n(%)]			0.232	0.972
≤30	2 (4.55)	2 (4.65)		
31–45	6 (13.64)	7 (16.28)		
46–59	23 (52.27)	23 (53.49)		
≥60	13 (29.55)	11 (25.58)		
Caregiver Gender [n(%)]			0.552	0.458
Male	17 (38.64)	20 (46.51)		
Female	27 (61.36)	23 (53.49)		
Relationship to Patient [n(%)]			0.197	0.978
Spouse	29 (65.91)	28 (65.12)		
Children	11 (25.00)	10 (23.26)		
Parents	3 (6.82)	4 (9.30)		
Siblings	1 (2.27)	1 (2.33)		
Marital Status [n(%)]			2.371	0.306
Unmarried	2 (4.55)	1 (2.33)		
Married	42 (95.45)	40 (93.02)		
Divorced/Widowed	0 (0.00)	2 (4.65)		
Religious Belief [n(%)]			0.001	0.977
No	41 (93.18)	40 (93.02)		
Yes	3 (6.82)	3 (6.98)		
Education Level [n(%)]			0.835	0.841
Junior high school or below	24 (54.55)	22 (51.16)		
High school/technical school	8 (18.18)	11 (25.58)		
Vocational college/associate degree	8 (18.18)	6 (13.95)		
Bachelor’s degree or above	4 (9.09)	4 (9.30)		
Employment Status [n(%)]			1.018	0.601
Employed	33 (75.00)	28 (65.12)		
Retired	5 (11.36)	7 (16.28)		
Unemployed	6 (13.64)	8 (18.60)		
Daily Caregiving Hours [n(%)]			7.146	0.067
1–3	5 (11.36)	1 (2.33)		
4–6	8 (18.18)	9 (20.93)		
7–9	6 (13.64)	1 (2.33)		
10–12	25 (56.82)	32 (74.42)		
Chronic Disease in Caregiver [n(%)]			0.023	0.879
No	15 (34.09)	14 (32.56)		
Yes	29 (65.91)	29 (67.44)		
Monthly Household Income per Capita (¥)			6.976	0.073
≤3000	14 (31.82)	20 (46.51)		
3001–6000	12 (27.27)	15 (34.88)		
6001–10000	17 (38.64)	6 (13.95)		
>10000	1 (2.27)	2 (4.65)		
Patient Age [n(%)]			3.409	0.333
Patient Age [n(%)]			3.409	0.333
31–45	3 (6.82)	7 (16.28)		
46–59	20 (45.45)	18 (41.86)		
≥60	20 (45.45)	15 (34.88)		
Patient Gender [n(%)]			0.119	0.730
Male	19 (43.18)	17 (39.53)		
Female	25 (56.82)	26 (60.47)		
Patient Self-Care Ability [n(%)]			3.317	0.190
Totally dependent	0 (0.00)	3 (6.98)		
Partially dependent	28 (63.64)	27 (62.79)		
Fully independent	16 (36.36)	13 (30.23)		
Primary Diagnosis [n(%)]			6.366	0.498
Lung cancer	19 (43.18)	19 (44.19)		
Esophageal cancer	3 (6.82)	5 (11.63)		
Gastric cancer	1 (2.27)	5 (11.63)		
Liver cancer	3 (6.82)	3 (6.98)		
Colorectal cancer	4 (9.09)	3 (6.98)		
Breast cancer	6 (13.64)	4 (9.30)		
Ovarian cancer	5 (11.36)	1 (2.33)		
Cervical cancer	3 (6.82)	3 (6.98)		
Health Insurance Type [n(%)]			0.323	0.570
Urban employee insurance	19 (43.18)	16 (37.21)		
Cooperative medical insurance	25 (56.82)	27 (62.79)		
Awareness of Diagnosis [n(%)]			1.079	0.583
Fully aware	7 (15.91)	6 (13.95)		
Partially aware	37 (84.09)	36 (83.72)		
Not aware at all	0 (0.00)	1 (2.33)		

Although there were no statistically significant differences in baseline demographic and clinical characteristics between the two groups (all *p* > 0.05), we noted that the p-values for monthly household income per capita (*p* = 0.073) and daily caregiving hours (*p* = 0.067) approached the conventional significance threshold. Given the plausible relevance of these variables to caregiver burden, we conducted a sensitivity analysis adjusting for income and caregiving hours in the linear mixed-effects model. The results remained consistent with the primary findings, suggesting that any potential baseline imbalance in these variables did not materially affect the estimated observed between-group differences. At baseline (T_0_), there were no statistically significant differences between the intervention and control groups in total caregiver burden, personal strain, or role strain scores (*p* > 0.05) (**Table 3**), indicating good comparability between groups. Although patients’ awareness of diagnosis was collected descriptively (**Table 2**), it was not included as a covariate or exploratory predictor in the primary analyses because the study was not designed or powered to examine its moderating effects. Accordingly, no causal or moderating inferences regarding this variable are drawn from the present data.

**Table 3:** Comparison of care burden and quality of life between the two groups before intervention.

Outcomes	Control group (n=44)	Intervention group (n=43)	*t*	*p*
Caregiver Burden	43.55 ± 5.88	44.14 ± 5.48	-0.487	0.627
Personal Strain	32.70 ± 4.86	33.26 ± 4.22	-0.564	0.574
Role Strain	10.84 ± 3.51	10.88 ± 3.79	-0.055	0.957
Quality of Life	61.14 ± 5.38	61.05 ± 4.93	0.081	0.935
Physical Domain	10.95 ± 1.18	10.88 ± 1.22	0.275	0.784
Psychological Domain	10.80 ± 1.53	10.67 ± 1.71	0.347	0.729
Social Relationships Domain	11.86 ± 1.55	12.00 ± 1.09	-0.474	0.637
Environmental Domain	10.57 ± 1.59	10.79 ± 1.83	-0.605	0.547

Repeated-measures ANOVA showed significant group × time interaction effects for total caregiver burden, personal strain, and role strain, indicating that changes in caregiver burden over time differed between the intervention and control groups (**Table 4**). The interaction effect sizes ranged from medium to large, with partial eta-squared values of 0.062 for personal strain, 0.120 for role strain, and 0.149 for total caregiver burden. In the intervention group, total caregiver burden decreased from 44.14 ± 5.48 at T_0_ to 37.02 ± 3.74 at T_1_ and 39.02 ± 3.89 at T_2_. Bonferroni-adjusted within-group comparisons showed that total caregiver burden and role strain remained significantly lower at both T_1_ and T_2_ than at baseline, whereas personal strain was significantly reduced at T_1_. For QOL outcomes, significant group × time interaction effects were observed for overall QOL and all four WHOQOL-BREF domains (**Table 4**). The interaction effect was large for the physical domain (*η²p* = 0.450), medium for the social relationships domain (*η²p* = 0.096) and environmental domain (*η²p* = 0.083), and small-to-medium for overall QOL (*η²p* = 0.057) and the psychological domain (*η²p* = 0.039). In the intervention group, overall QOL increased from 61.05 ± 4.93 at T_0_ to 65.05 ± 4.95 at T_1_ and remained higher than baseline at T_2_ (64.05 ± 4.26). Bonferroni-adjusted comparisons showed significant improvements from baseline to T_1_ in overall QOL and all four domains. However, from T_1_ to T_2_, the intervention group showed significant declines in the physical domain (13.86 ± 1.25 to 11.35 ± 1.56; mean change = −2.51), psychological domain (12.86 ± 1.54 to 11.56 ± 2.70; mean change = −1.30), and social relationships domain (12.72 ± 1.56 to 11.56 ± 2.70; mean change = −1.16).

**Table 4:** Comparison of caregiver burden and QOL scores at different time point between intervention group and control group.

Outcomes	Group	n	T_0_	T_1_	T_2_	Group effect		Time effect		Group × time interaction		
						*F_group_*	*p_group_*	*F_time_*	*p_time_*	*F_interaction_*	*p_interaction_*	*η²p*
Caregiver Burden	Control group	44	43.55 ± 5.88	45.30 ± 5.93	43.68 ± 6.40	F(1, 85)=40.99	<0.001	F(2, 170)=6.47	0.002	F(2, 170)=14.82	<0.001	0.149
	Intervention group	43	44.14 ± 5.48	37.02 ± 3.74^a^	39.02 ± 3.89^a^							
Personal Strain	Control group	44	32.70 ± 4.86	34.25 ± 4.56	33.70 ± 4.88	F(1, 85)=9.70	0.003	F(2, 170)=0.35	0.704	F(2, 170)=5.59	0.005	0.062
	Intervention group	43	33.26 ± 4.22	30.60 ± 3.44^a^	31.60 ± 3.50							
Role Strain	Control group	44	10.84 ± 3.51	11.05 ± 3.72	9.98 ± 3.69	F(1, 85)=34.55	<0.001	F(2, 170)=12.77	<0.001	F(2, 170)=11.59	<0.001	0.120
	Intervention group	43	10.88 ± 3.79	6.42 ± 2.42^a^	7.42 ± 1.48^a^							
Quality of Life	Control group	44	61.14 ± 5.38	61.66 ± 4.03	60.14 ± 5.42	F(1, 85)=12.18	<0.001	F(2, 170)=5.51	0.005	F(2, 170)=5.15	0.007	0.057
	Intervention group	43	61.05 ± 4.93	65.05 ± 4.95^a^	64.05 ± 4.26^a^							
Physical Domain	Control group	44	10.95 ± 1.18	9.68 ± 1.18a	9.95 ± 1.18^a^	F(1, 85)=116.04	<0.001	F(1.60,136)=19.67	<0.001	F(1.60,136)=69.47	<0.001	0.450
	Intervention group	43	10.88 ± 1.22	13.86 ± 1.25^a^	11.35 ± 1.56^b^							
Psychological Domain	Control group	44	10.80 ± 1.53	11.64 ± 1.35^a^	10.30 ± 2.36^b^	F(1, 85)=12.02	<0.001	F(1.65,141)=15.10	<0.001	F(1.65, 141)=3.47	0.042	0.039
	Intervention group	43	10.67 ± 1.71	12.86 ± 1.54^a^	11.56 ± 2.70^b^							
Social Relationships Domain	Control group	44	11.86 ± 1.55	10.86 ± 1.82^a^	11.80 ± 1.36^b^	F(1, 85)=6.89	0.010	F(2, 170)=0.47	0.616	F(2, 170)=9.07	<0.001	0.096
	Intervention group	43	12.00 ± 1.09	12.72 ± 1.56	11.56 ± 2.70^b^							
Environmental Domain	Control group	44	10.57 ± 1.59	10.18 ± 1.79	10.80 ± 1.36	F(1, 85)=16.42	<0.001	F(2, 170)=2.39	0.098	F(2, 170)=7.74	<0.001	0.083
	Intervention group	43	10.79 ± 1.83	12.16 ± 1.65^a^	11.37 ± 1.70							

T₀, before intervention; T_1_, immediately after intervention; T_2_, 3-month post-intervention follow-up.

*F_group,_ p_group,_ F_time_, p_time_, F_interaction_* and *p_interaction_* represent the overall repeated-measures ANOVA test statistics for each outcome and apply jointly to both the control and intervention group rows. For outcomes violating the sphericity assumption (i.e., Physical Domain and Psychological Domain in the full analysis), the *p*-values for time and interaction effects were adjusted using the Greenhouse–Geisser correction.

*η²p*, partial eta squared for the interaction effect.

Data are presented as mean ± SD. ^a^*p* < 0.05 vs T₀ (before intervention); ^b^*p* < 0.05 vs T_1_ (immediately after intervention).

Between-group comparisons further showed that, at both T_1_ and T_2_, the intervention group had significantly lower total caregiver burden and role strain than the control group, these differences remained significant in the *post hoc* FDR sensitivity analysis (**Table 5**). For personal strain, the between-group difference was significant at T_1_, but the T_2_ difference was only nominally significant in unadjusted analysis (*p* = 0.024) and was not robust in the *post hoc* Holm or Bonferroni sensitivity correction (Holm-adjusted *p* = 0.091; Bonferroni-adjusted *p* = 0.377; see **Supplementary Tables 1**, **2**). The between-group effect size for total caregiver burden was large at both T_1_ and T_2_. Descriptively, the mean ZBI total score in the intervention group decreased from the severe burden range at T_0_ (44.14 ± 5.48) to the moderate burden range T_1_ (37.02 ± 3.74) and remained within the moderate category at T_2_ (39.02 ± 3.89). This represented a reduction of 7.12 points at T_1_ and 5.12 points at T_2_ compared with baseline, suggesting that the statistically significant changes may be potentially clinically meaningful. For QOL, the intervention group showed significantly higher overall QOL and physical domain scores than the control group at both T_1_ and T_2_, these differences remained significant in the *post hoc* FDR sensitivity analysis (**Table 5**). The psychological domain showed significant improvement at T_1_, but the T_2_ difference was only nominally significant in unadjusted analysis (*p* = 0.023) and was not robust in the *post hoc* Holm or Bonferroni sensitivity correction (Holm-adjusted *p* = 0.091; Bonferroni-adjusted *p* = 0.365; see **Supplementary Tables 1**, **2**). Therefore, the psychological domain finding at T_2_ should be interpreted as suggestive rather than confirmatory. The largest between-group effect was observed in the physical domain at T_1_. The social relationships and environmental domains showed significant between-group differences at T_1_, with large effect sizes, but these differences were not statistically significant at T_2_. Specifically, the between-group difference at T_2_, was not significant for the social relationships domain (*p* = 0.608, Cohen’s *d* = -0.11) or the environmental domain (*p* = 0.085, Cohen’s *d* = 0.37).

**Table 5:** Between-group differences, effect sizes, and 95% confidence intervals at different time points.

Outcomes	Time	MD (95% CI)	Cohen's *d* (95% CI)	*p*
Caregiver Burden	T_1_	-8.27 (-10.39, -6.16)	-1.66 (-2.15, -1.18)	<0.001
	T_2_	-4.66 (-6.92, -2.40)	-0.88 (-1.32, -0.44)	<0.001
Personal Strain	T_1_	-3.65 (-5.37, -1.92)	-0.90 (-1.34, -0.46)	<0.001
	T_2_	-2.10 (-3.91, -0.29)	-0.49 (-0.92, -0.07)	0.024
Role Strain	T_1_	-4.63 (-5.97, -3.29)	-1.47 (-1.94, -0.99)	<0.001
	T_2_	-2.56 (-3.76, -1.35)	-0.90 (-1.35, -0.46)	<0.001
Quality of Life	T_1_	3.39 (1.46, 5.32)	0.75 (0.32, 1.19)	<0.001
	T_2_	3.91 (1.83, 5.99)	0.80 (0.36, 1.24)	<0.001
Physical Domain	T_1_	4.18 (3.66, 4.70)	3.45 (2.78, 4.12)	<0.001
	T_2_	1.39 (0.80, 1.98)	1.01 (0.56, 1.46)	<0.001
Psychological Domain	T_1_	1.22 (0.61, 1.84)	0.85 (0.41, 1.29)	<0.001
	T_2_	1.26 (0.18, 2.35)	0.50 (0.07, 0.93)	0.023
Social Relationships Domain	T_1_	1.86 (1.13, 2.58)	1.09 (0.64, 1.54)	<0.001
	T_2_	-0.24 (-1.16, 0.68)	-0.11 (-0.53, 0.31)	0.608
Environmental Domain	T_1_	1.98 (1.25, 2.71)	1.15 (0.70, 1.60)	<0.001
	T_2_	0.58 (-0.08, 1.23)	0.37 (-0.05, 0.80)	0.085

*p*-values shown are unadjusted for multiple comparisons. Multiplicity-adjusted analyses using the Benjamini-Hochberg FDR, Holm, and Bonferroni methods were conducted *post hoc* as sensitivity analyses to examine the robustness of the findings. Results that remained statistically significant after adjustment were interpreted as more robust, whereas findings significant only before adjustment were interpreted cautiously. See **Supplementary Tables 1**, **2** for full multiplicity-adjusted results.

T_1_, immediately after intervention; T_2_, 3-month post-intervention follow-up; MD, mean difference; CI, confidence interval.

Cohen’s *d* values of 0.2, 0.5, and 0.8 indicate small, medium, and large effect sizes.

Assumption testing and sensitivity analyses are presented in **Table 6**. Mauchly’s test indicated that the sphericity assumption was violated for the physical and psychological domains. Therefore, Greenhouse-Geisser corrections were applied to the time and group × time interaction effects for these outcomes. Residual normality was approximately satisfied for caregiver burden outcomes and overall QOL, while some QOL domains showed deviations from normality. Homogeneity of variance was violated for several caregiver burden outcomes and the social relationships domain. To examine the robustness of the findings, linear mixed-effects model sensitivity analyses were conducted using the same per-protocol complete-case sample. The group × time interaction effects remained statistically significant for all outcomes, consistent with the repeated-measures ANOVA results, and the LMM sensitivity analyses did not change the primary conclusions.

**Table 6:** Assumption testing for repeated-measures ANOVA and sensitivity analysis using a linear mixed-effects model.

Outcomes	Mauchly's *W*	*p*	G-G *ϵ*	Homogeneity of variance	Normality of residuals	LMM interaction *p*
Caregiver Burden	0.974	0.327	0.975	Violated	Approximate	<0.001
Personal Strain	0.994	0.772	0.994	Violated	Approximate	0.004
Role Strain	0.947	0.100	0.950	Violated	Approximate	<0.001
Quality of Life	0.988	0.592	0.988	Satisfied	Approximate	0.006
Physical Domain	0.751	<0.001	0.801	Satisfied	Deviated	<0.001
Psychological Domain	0.791	<0.001	0.827	Satisfied	Deviated	0.026
Social Relationships Domain	0.948	0.102	0.950	Violated	Deviated	<0.001
Environmental Domain	0.941	0.075	0.944	Satisfied	Deviated	<0.001

Mauchly’s *W*, Mauchly’s test statistic for sphericity; G-G *ϵ*, Greenhouse–Geisser epsilon correction factor; LMM interaction *p*, the *p*-values for the group × time interaction from the linear mixed-effects model. The LMM included group, time, and group × time interaction as fixed effects and participant as a random intercept. The model was fitted using the same per-protocol complete-case sample as the primary analysis; participants with missing outcome observations were not included.

To examine whether assessment modality at T_2_ influenced outcome reporting, we compared face-to-face (n=77) and WeChat video (n=10) assessments. The distribution of modalities was balanced between groups (*p* = 0.521). No significant differences were found between the two modalities across all outcome measures at T_2_ (unadjusted *p* = 0.437-0.967; adjusted for baseline and group, *p* = 0.220-0.954; FDR-adjusted *q* > 0.95). Group × modality interaction terms were all non-significant (*p* = 0.406-0.999). A sensitivity analysis excluding the 10 WeChat participants showed consistent results for 7 of 8 outcomes; the Psychological Domain changed from *p* = 0.043 to *p* = 0.056, which is consistent with its non-robustness under multiple comparison correction (Bonferroni-adjusted *p* = 0.342). These results suggest that assessment modality did not materially affect the study findings. Detailed results are presented in **Supplementary Tables 3**, **4**.

## Discussion

### Impact of cognitive behavioral interventions on caregiver burden in cancer caregivers

Caregiver burden may emanate from multiple dimensions. Caregivers experience considerable emotional strain, including anxiety, frustration, and anger, arising directly from caregiving tasks. CBI has been associated with equipping caregivers with skills to manage negative emotions more effectively, which may help mitigate emotional distress (31). Furthermore, caregivers frequently neglect their own physical health, manifesting as irregular eating patterns, sleep deprivation, and inadequate physical activity. CBI may assist caregivers in establishing healthier lifestyle routines, which could alleviate burden related to physical well-being. Social isolation, perceived lack of support, and interpersonal strain are also common challenges (32). CBI may facilitate the development of more robust social support networks, which could reduce social relationship burden (33). Additionally, caregivers face significant time constraints, dedicating extensive time and energy to caregiving at the expense of personal needs and interests. CBI may offer strategies for improved time management, potentially enabling caregivers to achieve a better balance between caregiving responsibilities and personal life and thereby reduce time-related pressure (34).

In this study, caregivers of patients with various cancer types were treated as a unified cohort. Although different cancer types present distinct physical trajectories, the event of cancer recurrence itself typically acts as a profound psychological stressor. The transition from clinical remission back to active disease often triggers comparable psychosocial challenges among caregivers, including disrupted hope, existential distress, and the abrupt resumption of demanding caregiving duties. Although recurrence may represent a shared psychological stressor across cancer types, differences in symptom burden, prognosis, and treatment trajectories may still influence caregivers’ experiences. Therefore, pooling caregivers across cancer types was a pragmatic approach in the present study, and the findings should be interpreted cautiously in light of this clinical heterogeneity.

Our findings suggest that structured CBI may be associated with reduced caregiver burden among family caregivers of patients with recurrent cancer over the immediate and 3-month follow-up period. Because group assignment was based on inpatient ward, possible ward-level influences could not be separated from the intervention condition, which limits causal interpretation of the observed between-group differences. The reduction in caregiver burden may be potentially clinically meaningful, as the mean ZBI total score in the intervention group decreased from the severe burden range at baseline to the moderate burden range after the intervention and remained within the moderate range at the 3-month follow-up. These observed differences were also supported by moderate-to-large effect sizes and the sensitivity analysis. Several components of the intervention provide plausible explanations for the observed differences in caregiver burden. Psychoeducation, cognitive restructuring, and coping-skills training were intended to help caregivers reappraise excessive responsibility, self-blame, and other maladaptive interpretations of caregiving stress. These components may have supported more adaptive responses to recurrent cancer-related demands. However, the total burden score increased slightly between T_1_ and T_2_, indicating partial attenuation of the immediate improvement during the 3-month follow-up. Given the limited follow-up period, it remains unclear whether the observed difference at T_2_ reflects short-term maintenance of intervention-associated changes, gradual attenuation, or changes in caregiving demands over time. Future studies with longer follow-up and repeated assessment of cognitive and coping processes are needed to clarify the trajectory and potential mechanisms of these changes. Periodic reinforcement sessions may also be evaluated as a strategy to support continued skill use after the structured intervention ends. However, the reduction in personal strain at T_2_ was not robust in the *post hoc* multiplicity-adjusted sensitivity analyses (see **Supplementary Tables 1**, **2**), suggesting that this subdomain is less certain and warrants further investigation.

### Impact of cognitive behavioral interventions on quality of life in cancer caregivers

The intervention group showed higher overall QOL and higher physical domain scores than the control group at both T_1_ and T_2_, and these differences remained significant in the *post hoc* FDR sensitivity analysis. For the psychological domain, although the intervention group showed higher scores at T_2_ in unadjusted analyses, this difference was not robust in the *post hoc* Holm or Bonferroni sensitivity correction (Holm-adjusted *p* = 0.091; see **Supplementary Tables 1**, **2**). Therefore, the psychological domain improvement at T_2_ should be interpreted with caution and considered exploratory rather than confirmatory. In contrast, the between-group differences in the social relationships and environmental domains were no longer statistically significant at T_2_. In addition, the significant declines in the physical, psychological, and social relationships domains from T_1_ to T_2_ suggest partial erosion of the immediate QOL gains over the 3-month follow-up, rather than simple stabilization. This may be attributed to the nature of the short-term intervention, which does not include continued follow-up support. This may be attributed to the nature of the short-term intervention, which does not include continued follow-up support. It is also noteworthy that the total caregiver burden score in the control group increased from T_0_ 43.55 ± 5.88 at to 45.30 ± 5.93 at T_1_. This pattern may reflect the increasing caregiving demands and psychological stress associated with the clinical course following cancer recurrence. Therefore, the observed between-group difference at T_1_ may have resulted not only from a reduction in caregiver burden in the intervention group, but also partly from an increase in burden in the control group over the same period. This suggests that the observed between-group differences should be interpreted as a relative group difference within the context of the evolving disease trajectory, rather than solely as an absolute observed reduction. Further studies incorporating detailed clinical indicators of disease progression are needed to clarify the extent to which changes in caregiver burden are attributable to the intervention itself versus changes in patients’ clinical condition. The significant fluctuations observed in several QOL domains in the control group suggest that caregiver quality of life may vary over time even in the absence of a structured psychological intervention. Possible explanations include changes in patients’ clinical condition and caregiving demands, contextual or seasonal influences, regression to the mean, and reactivity to repeated assessment. Because these factors were not systematically measured in the present study, their contribution to the observed fluctuations cannot be determined and should be examined in future research.

A meta-analysis of randomized controlled trials involving 2,348 informal cancer caregivers found that CBI reduced anxiety and depression, whereas the pooled improvement in QOL did not reach statistical significance (17). In addition, a couple-focused CBI involving patients with cervical cancer receiving chemotherapy and their caregivers reported short-term improvements in caregiver outcomes (35). Compared with previous studies, our study extends the existing evidence by focusing specifically on family caregivers of patients with confirmed cancer recurrence, a population facing distinctive psychological and caregiving challenges. However, given the non-randomized design and relatively short follow-up period, the current findings should be regarded as preliminary evidence that warrants further confirmation in larger randomized studies with longer follow-up.

Although intervention mechanisms were not directly evaluated, several components of the Cognitive Behavioral framework may provide plausible explanations for the observed QOL pattern. Cognitive restructuring and emotion-regulation exercises were intended to help caregivers reappraise excessive responsibility, self-blame, and threatening interpretations associated with recurrence. Behavioral activation, problem-solving, and self-care planning were designed to encourage the reintroduction of rest, physical activity, and personally meaningful activities into daily routines. Communication and support-seeking exercises were intended to facilitate constructive interactions with patients, family members, and healthcare professionals, while relaxation and breathing exercises provided practical strategies for managing acute stress. Because our study did not measure automatic thoughts, anxiety, depression, emotion regulation, coping, self-efficacy, self-care behavior, communication quality, perceived social support, or physiological arousal. Nor did it conduct a formal mediation analysis. The observed changes in QOL therefore cannot be attributed to any specific cognitive or behavioral pathway. Future studies should include prespecified mediator measures and evaluate whether changes in these processes statistically account for changes in caregiver burden or QOL.

The decline in several QOL domains between the immediate and 3-month assessments suggests partial attenuation of the early intervention-associated improvements. One possible explanation is that the structured intervention ended after eight sessions and did not include formal maintenance support. Continued caregiving demands may also have limited caregivers’ ability to practice the cognitive and behavioral strategies consistently. In addition, recurrent cancer can involve new symptoms, treatment changes, functional decline, or transition to palliative care, all of which may affect caregiver QOL independently of psychological intervention (15, 36). The lack of significant between-group differences in the social relationships and environmental domains at the 3-month follow-up may reflect the influence of factors that extend beyond an individually oriented psychological intervention. Social relationships are shaped by family communication, distribution of caregiving responsibilities, and access to informal support (16). The environmental domain may be affected by financial pressure, healthcare access, community services, transportation, housing, and social-welfare resources. A research has shown that social support, diagnosis disclosure, and culturally shaped communication practices are associated with caregiver burden (25). These contextual factors may limit the extent to which individual cognitive and behavioral skills translate into sustained changes in social or environmental QOL. In the context of Chinese culture, caregiving is often shaped by cultural expectations of filial responsibility and family obligation, which may influence caregivers’ willingness to seek help, express distress, or prioritize self-care. Therefore, intervention components related to help-seeking, boundary setting, and self-care may require cultural adaptation in settings with different caregiving norms (22–24). Healthcare infrastructure may also affect intervention feasibility, as access to psychological services, community support, and referral pathways varies across systems. In Western healthcare settings, CBI may be integrated into existing psychosocial oncology and caregiver-support services. However, differences in service organization, insurance coverage, and family involvement in care may influence caregivers’ needs and intervention responses. Thus, although the core Cognitive Behavioral principles may be broadly applicable, the delivery format and cultural framing should be adapted to local healthcare structures and caregiving norms. However, because our study did not include diagnosis disclosure as a formal variable in the outcome analyses, these cultural and communication-related considerations are offered as contextual interpretation rather than as empirical findings from the present data.

In summary, structured CBI may be associated with favorable immediate and 3-month follow-up outcomes in the caregiving context of cancer recurrence. However, the sustainability of these effects remains uncertain because of the relatively short follow-up period. Because the intervention group and control group were recruited from different wards, with one ward per study arm, the observed between-group differences should not be interpreted as isolating the specific effect of CBI from possible ward-level influences. This ward-based allocation design limits the identifiability of CBI-specific associations and the causal interpretation of the findings. Future studies should consider a multi-phase intervention model that incorporates periodic booster sessions, telehealth monitoring, or digital follow-up support after the initial CBI program to reinforce acquired skills and promote the maintenance of observed gains. Incorporating family member involvement and leveraging community resources may help build a more robust and supportive caregiving ecosystem. In addition, stratified intervention approaches based on caregivers’ individual characteristics and needs should be developed to increase responsiveness. Further well-designed studies with extended follow-ups and comprehensive outcome evaluations are needed to further examine the associations of CBI with caregiver burden and quality of life over immediate and 3-month follow-up periods, which may inform the refinement of intervention protocols and facilitate their integration into routine oncology care.

### Limitations

First, the sample size was relatively small, and the study was conducted as a non-randomized quasi-experimental study at a single center with participants drawn exclusively from the Chinese population. These factors may limit the generalizability of our findings. Future multi-center studies with larger and more diverse samples are warranted to validate the effectiveness of CBI in different cultural and healthcare contexts. Moreover, outcome assessors were not blinded to group allocation. Given that the ZBI and WHOQOL-BREF were self-reported measures and were administered with assistance from study staff when needed, potential detection bias and inflation of the estimated observed between-group differences cannot be excluded. In addition, the intervention group received substantially more structured contact with healthcare professionals than the control group. Because no attention-control condition was included, the observed between-group differences may partly reflect nonspecific effects of additional attention, social interaction, or support rather than CBI-specific effects alone. Therefore, the findings should be interpreted as associations rather than definitive evidence of a CBI-specific causal effect. Second, because group allocation was ward-based rather than truly randomized, we did not stratify participants by cancer type or clinical stage, but included all patients as a single group. Different cancers may follow distinct recurrence trajectories, carry varying prognostic implications, and impose different physical symptom burdens. Although baseline comparisons revealed no significant differences between the two wards in either the distribution of primary diagnoses or overall caregiver burden scores, the absence of true randomization means that subtle, unmeasured clinical imbalances may still have been present. For instance, the specific symptom distress associated with different cancers, such as progressive respiratory compromise in lung cancer versus nutritional deterioration in gastric cancer, places qualitatively different demands on caregivers. Such latent clinical differences could have implicitly influenced the nature of baseline burden in ways not fully captured by aggregate scores. Furthermore, we did not collect longitudinal data on patients’ disease progression during the follow-up period. The failure to account for this dynamic clinical confounder limits our ability to disentangle the true long-term effects of the intervention from the compounding stress of ongoing patient decline. As a result, the applicability of our findings to specific cancer populations may be limited. Future research should aim to develop and evaluate tailored interventions targeting particular cancer types and stages. Furthermore, allocation was ward-based rather than randomized, so residual unmeasured confounding and contamination bias could not be fully excluded. As the intervention and control groups were recruited from distinct single wards, ward-level factors—including ward culture, staffing, nursing routines, patient mix, caregiver education, and standard care delivery—were fully confounded with group assignment and inseparable from the observed between-group differences. This represents a fundamental design limitation affecting identifiability, internal validity, and causal inference, rather than merely a statistical modeling limitation. Third, the follow-up assessment was conducted only three months after the intervention, which is a relatively short timeframe for evaluating long-term outcomes. In addition, specific occupational information, including whether caregivers were healthcare workers (HCWs), was not collected in this study. This may have limited our ability to account for the potential influence of medical knowledge, occupational stress, and dual professional–family caregiving roles on caregiver burden and quality of life. Future studies should collect detailed occupational information and consider HCW status as a potential confounder or effect modifier. Longer follow-up periods are recommended in future studies to more comprehensively assess the sustained effects of CBI on caregiver burden and quality of life. Fourth, although attendance and homework completion rates were high, we did not systematically assess intervention feasibility or acceptability using standardized scales or qualitative interviews. Consequently, the observed high adherence should not be interpreted as formal evidence of feasibility or acceptability. Future implementation studies should incorporate formal mixed-methods evaluations of these implementation outcomes. Fifth, the intervention was delivered in small groups of 5–8 participants with a limited number of facilitators, which may have introduced clustering effects—i.e., participants within the same intervention group may have been more similar to each other than to participants in other groups due to shared facilitator characteristics and group dynamics. Because the study was not designed to estimate group-level random effects and the number of intervention groups was limited, we were unable to include group-level clustering in the statistical models. As a result, standard errors may be underestimated. Future studies with larger numbers of groups should consider multilevel modeling to account for this nested structure. Because group assignment was determined by ward, ward-level clustering and ward-level confounding could not be reliably estimated or separated from group assignment. Therefore, standard errors may have been underestimated, and the findings should be interpreted cautiously.

## Conclusions

The findings of this study suggest that structured CBI may be associated with reductions in total caregiver burden and role strain, as well as improvements in overall QOL and the physical domain, over a 3-month follow-up period. However, given the ward-based allocation, these findings should be interpreted cautiously as preliminary associations, as ward-level influences could not be separated from the intervention condition. In addition, improvements in the psychological domain and personal strain at 3 months were not robust in *post hoc* conservative multiple-adjusted sensitivity analyses. These secondary findings should therefore be interpreted as exploratory and hypothesis-generating, requiring confirmation in future studies with pre-specified outcomes and larger samples. Further large-scale, multi-center studies with extended follow-up periods are needed to examine the sustainability of these effects and to evaluate the intervention across diverse populations and healthcare settings. In addition, individual differences among caregivers should be considered to inform the development of more personalized and targeted interventions.

## Data Availability

The raw data supporting the conclusions of this article will be made available by the authors, without undue reservation.
