# Supplementary material for: The association of cognitive behavioral intervention with caregiver burden and quality of life among family caregivers of cancer recurrence patients
Source: Front Psychiatry. 2026 Oct 15;17:1904821. doi: 10.3389/fpsyt.2026.1904821 (PMC13716778; doi:10.3389/fpsyt.2026.1904821)
Supplement: Supplementary file 1 [file DataSheet1.pdf]

Supplementary Statistical Reanalysis Report

The Effect of Cognitive Behavioral Intervention on Caregiver Burden and Quality of Life Among Family Caregivers of Cancer Recurrence Patients

1. Multiple Comparison Correction

The original manuscript conducted multiple significance tests across 8 outcome measures (total caregiver burden, personal strain, role strain, total quality of life, and the physical, psychological, social relationships, and environmental domains) at 3 time points without adjustment for multiplicity. In this revision, we applied Bonferroni, Holm, and Benjamini–Hochberg (BH) false discovery rate (FDR) corrections according to testing families. Family A comprised the group × time interaction effects for the 8 outcomes (the primary inference of the study, 8 tests); Family B comprised the between-group comparisons at T_1 and T_2 for the 8 outcomes (16 tests); and Family C comprised the within-group pairwise comparisons across time points, for which Bonferroni correction had already been applied in the original analysis and was therefore maintained unchanged.

Table S1. Multiple comparison correction results for group × time interaction effects (Family A, 8 tests)

Outcomes	*F*	η^2p	*p*	Adjusted *p* / *q*		
				Bonferroni	Holm	BH-FDR
Caregiver Burden	14.82	0.149	<0.001	<0.001	<0.001	<0.001
Personal Strain	5.59	0.062	0.004	0.036	0.013	0.006
Role Strain	11.59	0.120	<0.001	<0.001	<0.001	<0.001
Quality of Life	5.15	0.057	0.007	0.054	0.013	0.008
Physical Domain	69.47	0.450	<0.001	<0.001	<0.001	<0.001
Psychological Domain	3.47	0.039	0.043	0.342	0.043	0.043
Social Relationships Domain	9.07	0.096	<0.001	0.001	<0.001	<0.001
Environmental Domain	7.74	0.083	<0.001	0.005	0.002	<0.001

η^2p = partial eta-squared; BH-FDR = Benjamini–Hochberg false discovery rate. Bonferroni- and Holm-adjusted values are adjusted *p* values, whereas BH-FDR-adjusted values are *q* values.

Table S2. Multiple comparison correction results for between-group comparisons at each time point (Family B, 16 tests)

Outcomes	Time	MD	95%CI	*d*	*p*	Adjusted *p* / *q*		
						Bonferroni	Holm	BH-FDR
Caregiver Burden	T_1	-8.27	(-10.39,-6.16)	-1.66	<0.001	<0.001	<0.001	<0.001
Caregiver Burden	T_2	-4.66	(-6.92,-2.40)	-0.88	<0.001	0.002	<0.001	<0.001
Personal Strain	T_1	-3.65	(-5.37,-1.92)	-0.90	<0.001	0.001	<0.001	<0.001
Personal Strain	T_2	-2.10	(-3.91,-0.29)	-0.49	0.024	0.377	0.091	0.027
Role Strain	T_1	-4.63	(-5.97,-3.29)	-1.47	<0.001	<0.001	<0.001	<0.001
Role Strain	T_2	-2.56	(-3.76,-1.35)	-0.90	<0.001	0.001	<0.001	<0.001
Quality of Life	T_1	3.39	(1.46,5.32)	0.75	<0.001	0.012	0.004	0.001
Quality of Life	T_2	3.91	(1.83,5.99)	0.80	<0.001	0.005	0.002	<0.001
Physical Domain	T_1	4.18	(3.66,4.70)	3.45	<0.001	<0.001	<0.001	<0.001
Physical Domain	T_2	1.39	(0.80,1.98)	1.01	<0.001	<0.001	<0.001	<0.001
Psychological Domain	T_1	1.22	(0.61,1.84)	0.85	<0.001	0.003	0.001	<0.001
Psychological Domain	T_2	1.26	(0.18,2.35)	0.50	0.023	0.365	0.091	0.027
Social Relationships Domain	T_1	1.86	(1.13,2.58)	1.09	<0.001	<0.001	<0.001	<0.001

Outcomes	Time	MD	95%CI	*d*	*p*	Adjusted *p* / *q*		
						Bonferroni	Holm	BH-FDR
Social Relationships Domain	T_2	-0.24	(-1.16,0.68)	-0.11	0.608	1.000	0.608	0.608
Environmental Domain	T_1	1.98	(1.25,2.71)	1.15	<0.001	<0.001	<0.001	<0.001
Environmental Domain	T_2	0.58	(-0.08,1.23)	0.37	0.085	1.000	0.170	0.091

Note: MD = mean difference (intervention group − control group), using Welch's t-test with correction for unequal variances. *d* = Cohen's *d*. T_1 = immediately post-intervention; T_2 = 3-month follow-up. Bonf. = Bonferroni-adjusted p-value; Holm = Holm stepwise-adjusted p-value; FDR *q* = Benjamini–Hochberg-adjusted *q*-value.

Conclusions:

(1) All 8 interaction effects in Family A remained statistically significant after BH-FDR and Holm correction (all $q < 0.05$), indicating that the primary conclusions were not affected by multiplicity. Only under the most conservative Bonferroni correction did the Psychological Domain (adjusted $p = 0.342$) and overall Quality of Life (adjusted p = 0.054) no longer reach statistical significance.

(2) Of the 16 between-group comparisons in Family B, 14 were originally significant. After BH-FDR correction, all 14 remained significant. Under Holm/Bonferroni correction, Personal Strain at T_2 (Holm-adjusted $p = 0.091$) and Psychological Domain at T_2 (Holm-adjusted $p = 0.091$) fell below the significance threshold, while the remaining 12 comparisons remained robust. Social Relationships Domain and Environmental Domain at T_2 were non-significant both before and after correction, consistent with the original description.

(3) We suggest that the main text should state that the group × time interaction effects served as the primary inferential basis, with BH-FDR applied to control the false discovery rate; between-group comparisons at each time point are presented as secondary/exploratory analyses, with both original *p*-values and adjusted q-values reported. Accordingly, the "between-group advantages of the intervention for Personal Strain and Psychological Domain at 3-month follow-up" should be interpreted as attenuated effects that are not robust under strict correction, rather than as confirmatory conclusions.

3. Sensitivity Analysis of T_2 Assessment Modality

Among the 87 participants, 77 (88.5%) completed the T_2 assessment face-to-face, and 10 (11.5%) completed it via WeChat video call due to seeking medical care elsewhere or mobility difficulties. The distribution was balanced between groups: control group, 40 face-to-face (90.9%) and 4 WeChat (9.1%); intervention group, 37 face-to-face (86.0%) and 6 WeChat (14.0%). The difference between groups was not statistically significant (Fisher's exact test, p = 0.521; $\chi^2 = 0.505$, $p = 0.477$; Fisher's exact test was used as the minimum expected frequency was 4.94), indicating that assessment modality did not introduce systematic bias in between-group comparisons.

No statistically significant differences were found between the two assessment modalities in terms of caregiver demographic characteristics (gender, relationship to patient, marital status, education level, employment status, history of chronic disease, patient self-care ability, and patient awareness of diagnosis; all p > 0.09). Furthermore, no significant differences were observed between the two modalities for any outcome measure at baseline (T_0) or immediately post-intervention (T_1) (all $p > 0.05$; FDR-adjusted $q > 0.66$), suggesting that the two modality groups were comparable prior to the follow-up assessment.

Table S3. Comparison of outcomes between the two T_2 assessment modalities (full sample)

Outcomes	T_2 score（M±SD）		Unadjusted comparison		ANCOVA (adjusted for baseline and group)		
	Face-to-face (n=77)	WeChat (n=10)	*d*	*p*	*β* (95%CI)	*p*	$p_{interaction}$
Caregiver Burden	41.44±6.03	40.90±3.35	0.09	0.673	0.10 (-3.50,3.71)	0.954	0.912
Personal Strain	32.66±4.57	32.70±2.36	-0.01	0.967	0.24 (-2.64,3.13)	0.867	0.890
Role Strain	8.78±3.20	8.20±2.15	0.19	0.465	-0.24 (-2.16,1.67)	0.803	0.999
Quality of Life	62.12±5.40	61.70±3.92	0.08	0.768	-1.25 (-4.44,1.95)	0.440	0.926
Physical Domain	10.58±1.47	11.10±2.02	-0.33	0.454	0.57 (-0.35,1.48)	0.220	0.406
Psychological Domain	10.95±2.46	10.70±3.62	0.09	0.838	-0.41 (-2.08,1.26)	0.627	0.664
Social Relationships Domain	11.73±2.19	11.30±1.49	0.20	0.437	-0.45 (-1.90,1.00)	0.541	0.782
Environmental Domain	11.12±1.56	10.80±1.55	0.20	0.555	-0.45 (-1.47,0.57)	0.381	0.885

Note: *β* represents the adjusted mean difference for the WeChat video group relative to the face-to-face group. The ANCOVA model was specified as T_2 outcome ~ corresponding baseline value + group + assessment modality, with degrees of freedom (1, 83). The interaction p-value represents the p-value for the group × assessment modality interaction term added to the above model, with degrees of freedom (1, 82). *d* = Cohen's *d*. Given the small sample size in the WeChat group, Mann–Whitney U tests were additionally performed for confirmation, and the results were consistent (all $p > 0.32$). All p-values remained non-significant after FDR correction (all $q > 0.95$).

Table S4. Sensitivity analysis excluding WeChat video participants (group × time interaction effects)

Outcomes	Full sample (n=87)		Face-to-face assessment (n=77)		Consistency of conclusion
	F	*p*	*F*	*p*	
Caregiver Burden	14.82	<0.001	12.64	<0.001	Yes
Personal Strain	5.59	0.004	5.55	0.005	Yes
Role Strain	11.59	<0.001	9.02	<0.001	Yes
Quality of Life	5.15	0.007	5.57	0.005	Yes
Physical Domain	69.47	<0.001	62.89	<0.001	Yes
Psychological Domain	3.47	0.043	3.11	0.056	No
Social Relationships Domain	9.07	<0.001	8.75	<0.001	Yes
Environmental Domain	7.74	<0.001	7.92	<0.001	Yes

Note: The face-to-face only subsample comprised 40 participants in the control group and 37 in the intervention group. F represents the group × time interaction effect, with degrees of freedom (2, 170) for the full sample and (2, 150) for the face-to-face only subsample.

Conclusions:

(1) The distribution of assessment modalities was balanced between groups (Fisher's exact test, $p = 0.521$), indicating that assessment modality did not constitute a source of systematic bias in between-group comparisons.

(2) No statistically significant differences were found between the two assessment modalities across all T_2 outcomes: unadjusted *p*-values ranged from 0.437 to 0.967; after adjusting for baseline and group assignment, *p*-values ranged from 0.220 to 0.954, with all FDR-adjusted $q > 0.95$; the group × assessment modality interaction terms were also all non-significant (p = 0.406 to 0.999), indicating that modality effects did not differ between

groups. The absolute values of adjusted mean differences were all within 1.3 points, and all 95% confidence intervals included 0.

(3) After excluding the 10 WeChat video participants and repeating the primary analysis, 7 of the 8 outcomes remained consistent in significance, with no substantial changes in effect sizes. The only exception was the Psychological Domain, for which the *p*-value changed from 0.043 (full sample) to 0.056 (face-to-face only), crossing the 0.05 threshold. Notably, this outcome also lost significance under Bonferroni correction in Family A (adjusted $p = 0.342$) and had the smallest effect size among all outcomes ($\eta^2 p = 0.039$). Therefore, the intervention effect on the Psychological Domain should be interpreted as "suggestive but not robust," a conclusion that is consistent with, rather than additional to, the multiple comparison correction results.

(4) Power considerations: With sample sizes of 77 (face-to-face) and 10 (WeChat), at $\alpha = 0.05$, the study had 80% power to detect effects of Cohen's $d \approx 0.95$ or larger, and only 31% power to detect medium effects ($d = 0.5$). Therefore, the above results should be interpreted as "no evidence was found that assessment modality influenced outcomes," rather than "the two modalities were confirmed to be equivalent."
